# The combined effect of probiotic cultures and incubation final pH on the quality of buffalo milk yogurt during cold storage

**DOI:** 10.1002/fsn3.580

**Published:** 2017-12-21

**Authors:** Abdullah Akgun, Fehmi Yazici, Haci Ali Gulec

**Affiliations:** ^1^ Engineering Faculty Department of Food Engineering Trakya University Edirne Turkey; ^2^ Engineering Faculty Department of Food Engineering Ondokuz Mayis University Samsun Turkey

**Keywords:** buffalo milk yogurt, cold storage, incubation pH, starter culture

## Abstract

The combined effects of starter culture type (SCT) and incubation final pH (IFpH) on the physicochemical and organoleptic properties of buffalo milk yogurt containing 3 g·100 g^−1^ milk fat were investigated throughout 20 days of storage at 4°C. The postacidification kinetics fitted to zero‐order reaction for all buffalo milk yogurt samples. The reaction rate constants of the buffalo milk yogurt samples containing YC‐X11, ABY‐2, and ABT‐4 cultures were 0.010, 0.007, and 0.004 g·100 g^−1^·day^−1^, respectively. Regardless of the IFpH, the absence of *Lactobacillus delbrueckii* subsp. *bulgaricus* in the starter culture increased the syneresis. L*, a*, and b* values were not affected by the IFpH and the SCT. ABY‐2 culture increased the amount of organic acids during cold storage in comparison with the YC‐X11, while its effect on the proportions of saturated and unsaturated fatty acids was not significant. The results of sensory evaluation revealed that a more acceptable buffalo milk yogurt can be manufactured by using probiotic ABY‐2 culture.

## 1 INTRODUCTION

Final composition and physicochemical and sensory aspects of a fermented milk product such as yogurt are influenced mainly by chemical composition of milk (Akgun, Yazici, & Gulec, 2016) and processing conditions (Nguyen, Ong, Kentish, & Gras, 2014). The effects of milk composition on the mentioned properties of buffalo milk yogurt are well described in our previous study (Akgun et al., 2016). Starter culture type (SCT) and incubation final pH (IFpH) are considered as other important factors which affect overall quality of buffalo milk yogurt during cold storage.

*Streptococcus thermophilus* and *Lactobacillus delbrueckii* subsp. *bulgaricus* are typical strains in yogurt manufacturing. Yogurt that contains probiotic bacteria such as *Lactobacillus acidophilus* and *Bifidobacteria* (Tamime & Robinson, 1999) is popular in dairy industry due to their health‐promoting properties (Ravula & Shah, 1998). Also, a wide range of products with different flavors, textures, and consistencies were obtained by mixed strains of these microorganisms (Mckinley, 2005). Olson and Aryana (2008) stated that an excessively high inoculated level of *L. acidophilus* prolonged the incubation time and resulted in an inferior quality in cow milk yogurt. Up to now, other studies on production of bioyogurt have mainly focused on evaluating the viability of probiotic bacteria in fermented milk products and their potential health benefits (Maragkoudakis et al., 2006). However, to the best of our knowledge, there is no comprehensive study on the evaluation of the effects of SCT and IFpH on the physicochemical and organoleptic properties of buffalo milk yogurt during cold storage. The process variables affecting the gel formation and syneresis in buffalo milk yogurt are also not well understood.

The combined assessment of organic and fatty acid profiles in yogurt with the activity of different starter cultures is a key issue to identify the effect of SCT on the flavor and aroma characteristics of the final product. The organic acid profile in a fermented dairy food is an indicator of the metabolic activity of added bacterial cultures (Maragkoudakis et al., 2006). Vénica, Perotti, and Bergamini (2014) reported that there was difference between organic acid profiles of yogurt samples having similar population of starter cultures, which was attributed to the changes in matrix structure of yogurt. Moreover, organic acids play an important role as natural preservatives in yogurt and they also affect the final characteristics of the product and cell viability of probiotics (Shah, 2017). In this context, Cruz et al. (2013) stated that the diversity of proteolytic activity of probiotic cultures caused production of additional organic acids which leads to more complex yogurt structure. The effect of SCT on the distribution of organic acids in buffalo milk yogurt was not explained enough in the literature. Fatty acids have also important role in the organoleptic and nutritional qualities of milk products. Especially, short‐chain fatty acids are involved in developing the sensory quality of products (Beshkova, Simova, Frengova, & Simov, 1998). It showed that yogurt is a good source of *n* − 3 fatty acids and the change in fatty acids during cold storage depends on milk source (Serafeimidou, Zlatanos, Kritikos, & Tourianis, 2013). Jia, Chen, Chen, and Ding (2016) stated that fatty acid profile of goat milk yogurt strictly depends on amount of carbohydrates and the ratio of starter cultures. Naydenova, Iliev, and Mihaylova (2013) showed that yogurt manufacturing increased the C18:3 fatty acid percentage of the total fatty acids in comparison with the ratio in raw buffalo milk, while it decreased the omega‐6 to omega‐3 fatty acid ratio. However, the effects of processing conditions and cold storage on the fatty acids content of buffalo milk yogurt are not well described.

SCT may also have an effect on the acidification kinetics during yogurt manufacturing. The final textural and organoleptic properties of the yogurt product are also affected by the IFpH. The interactions between whey protein and casein micelles due to the level of IFpH may result in the formation of different gel structure. Improper texture, acidity, and hardness which reduce the acceptability of the final product may occur due to the extent of IFpH.

In this study, the main objectives are (1) to determine the combined effects of SCT and IFpH on the physicochemical and organoleptic properties of buffalo milk yogurt and (2) to investigate the biochemical and sensory changes during cold storage of buffalo milk yogurt comparatively. The effects of SCT on the organic acid and fatty acid profiles of buffalo milk yogurt were also evaluated in detail.

## 2 MATERIALS AND METHODS

### 2.1 Materials

Buffalo milk was obtained from farmers in Kizilirmak Delta in Bafra, Turkey. Plain starter cultures were obtained from Peyma Chr. Hansen, Istanbul, Turkey. Freeze‐dried (FD‐DVS) plain starter culture (YC‐X11) consisting of *S. thermophilus* and *L. delbrueckii* subsp. *bulgaricus* and probiotic starter cultures (ABY‐2 and ABT‐4) consisting of *Bifidobacterium* BB‐12^®^, *L. acidophilus* LA‐5^®^, *S. thermophilus*, and *L. delbrueckii* subsp*. bulgaricus*, and *Bifidobacterium* BB‐12^®^, *L. acidophilus* LA‐5^®^, and *S. thermophilus*, respectively, were obtained from Peyma Chr. Hansen, Istanbul, Turkey.

### 2.2 Yogurt manufacturing

**Figure 1:**
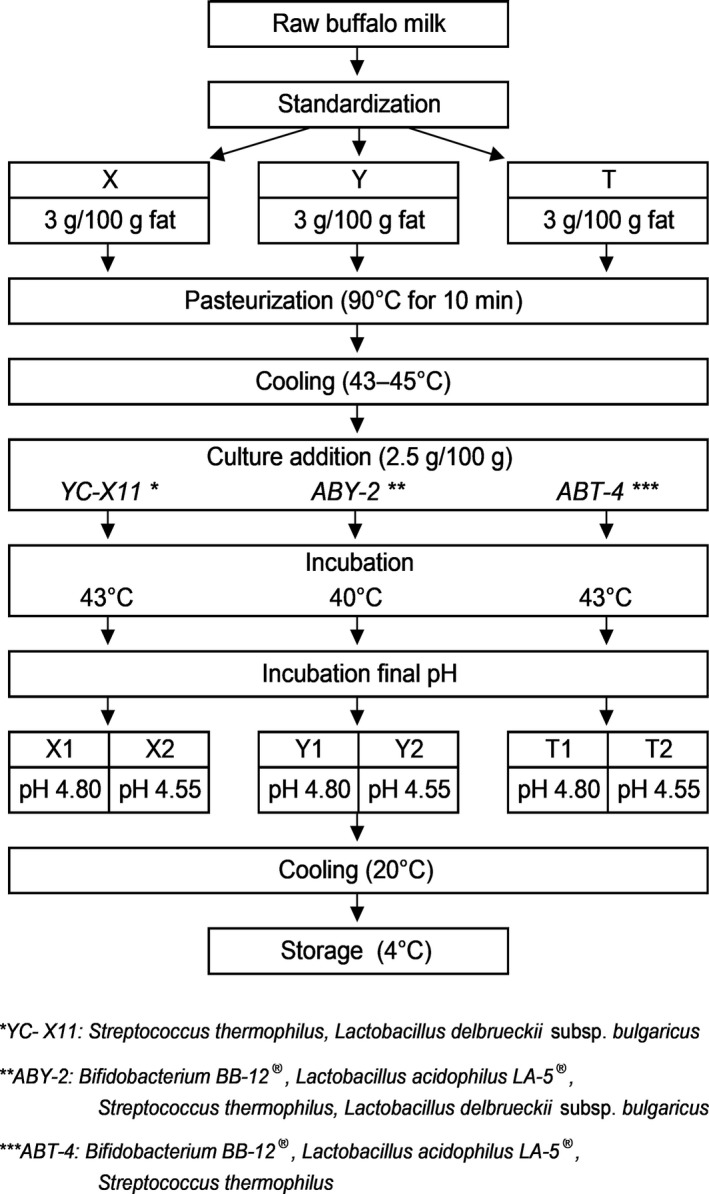
Process for manufacturing buffalo milk yogurt samples

The manufacturing procedures for the three different buffalo milk yogurt samples are shown in Figure 1. The milk was standardized to 3 g/100 g fat ratio by the addition of the removed cream to the raw milk using the Pearson square calculation. The mixtures were poured into 200 g polystyrene yogurt cups. Yogurt‐making procedure was replicated three times to a total of 18 vats.

### 2.3 Physicochemical analyses

The pH of the buffalo milk yogurt samples was measured during fermentation and 20 days of cold storage using a pH meter (Eutech CyberScan pH 100, Singapore). Total solids, fat, protein, ash, and titratable acidity were analyzed according to the methods described by Bradley et al. (1992) and AOAC (2000).

#### 2.3.1 Viscosity

The viscosity of buffalo milk yogurt was determined following the method described previously (Akgun et al., 2016) using a Brookfield viscometer (Model DV‐1+; Brookfield Engineering Laboratories, Inc., MA, USA). The viscometer was operated at 10 rpm (spindle number 3). Each result was recorded in cP after 20 s of rotation. The average value of three measurements was taken.

#### 2.3.2 Syneresis

Syneresis was measured according to the method described by Wacher‐Rodarte et al. (1993). Five milliliter of yogurt sample was centrifuged at 2,208*g* for 20 min at 4°C, and the separated whey was measured after 1 min. The syneresis rate (%) was expressed as the volume of separated whey per 100 g of yogurt. The analyses were carried out in triplicate.

#### 2.3.3 Color

L*, a*, and b* values of buffalo milk yogurt were determined using a sensing Chroma Meter (Konica Minolta CR‐400 Series, Japan). Prior to measurement, the instrument was calibrated with its white reference tile. Then, three readings were taken from the surface of each buffalo milk yogurt, and finally the average value was used.

#### 2.3.4 Organic acids analysis

The HPLC method described by Fernandez‐Garcia and McGregor (1994) was carried out for the analysis of organic acids with some following modifications. Yogurt samples (5 g) were weighed into 30 ml Teflon centrifuge tubes and diluted to 25 ml by 0.01 N H_2_SO_4_. The tubes were mixed for 1 min on a vortex mixer. Resulting mixtures were centrifuged at 7000 g for 7 min at 5°C in a refrigerated centrifuge (Sigma 3K30, Germany). Supernatant fractions were filtered through 0.45‐μm nylon filter (Supelco Iso‐Disc^™^, N‐25‐4 Nylon, 25 mm) into an HPLC vial.

The separation of organic acid was achieved using Shimadzu UFLC system (Shimadzu Corporation, Kyoto, Japan) equipped with ODS‐3 column (Shim‐pack, 150 × 4.6 mm). Twenty microliter of filtered supernatant fractions was injected. Organic acid was separated isocratically at 0.7 ml/min, at 65°C, using 10 mmol/L H_2_SO_4_ as the mobile phase. Lactic, acetic, formic, oxalic, citric, succinic, propionic, and butyric acids were detected at 210–280 nm with PDA detector (SPD‐M20A, Shimadzu, Japan). Individual organic acids were identified and quantified using external standards (Sigma Aldrich, Supelco, St. Louis, USA). Quantification of organic acids was performed from the standard curves obtained using five‐point solutions of predetermined concentrations. Triplicate extractions and injections were conducted for each sample.

#### 2.3.5 Fatty acids analysis

Fatty acid composition was determined after methylation (IUPAC, 1990) by a Shimadzu gas chromatograph (ModelGC‐2010, Shimadzu Corporation, Kyoto, Japan) using a Optima^®^ FAP column (60 m × 0.25 mm I.D., 0.25 μm) (Macherey‐Nagel, Düren, Germany). The temperatures of the injector port and detector were held at 260°C and 280°C, respectively. The injection volume was 1 μl. The carrier gas was helium at a pressure of 150 kPa. The split used was 1:100. The temperature of the column was held at 140°C for 2 min, raised to 170°C at 10°C/min and 215°C at 10°C/min, held at 215°C for 5 min, raised again to 230°C at 5°C/min and held at 230°C for 5 min, and finally raised to 240°C at 10°C/min and held at 240°C for 35 min. The identification of fatty acids was carried out by comparing their retention times with that of a standard mixture of fatty acids (Supelco 37 Component FAME Mixture, Cat. No. 18919‐1 Amp, St. Louis, MO, USA). The final concentration of fatty acids was reported as mg/g fat. All analyses were performed in triplicate.

### 2.4 Sensory analysis

Buffalo milk yogurt samples were organoleptically examined by a group of 15 panelists at the University of Ondokuz Mayis, Food Engineering Department, according to the method modified from Bodyfelt, Tobias, and Trout (1988), with maximum scores of 10, 5, 5, and 10 for flavor, body and texture, appearance and color, and general acceptability, respectively. The highest and the lowest numbers indicate liking extremely and disliking extremely, respectively. Initially, the panelists were trained in 2‐hr sessions prior to evaluation to be familiar with attributes and scaling procedures of yogurt samples. All buffalo milk yogurt samples were coded with three‐digit random numbers and presented to the panelists on a tray in individual booths. Orders of serving were completely randomized. Panelists were structured to cleanse their palate with plain crackers and water before tasting each sample.

### 2.5 Kinetic data analyses

The kinetic data analyses were done according to the kinetic model suggested by Fogler (2010). It was mentioned that the integral method uses a trial and error procedure to find reaction order. In the integral method, the reaction order was guessed and the differential equation used to model the batch system was integrated (Equation 1)

(1) $$\frac{dC}{dt}=\;\pm \;\;kC^{n}$$

where *C* is the concentration of the substance, *k* the reaction rate constant, and *n* the reaction order.

For example, integrating with *C* = *C*_0_ at *t* = 0,

Zero order; *C* = *C*_0_ ± *kt* was obtained and a plot of the concentration of a substance as a function of time will be linear with slope (±*k*) for a zero‐order reaction carried out in a constant volume batch reactor.

### 2.6 Statistical analysis

Statistical analysis of the data for the effects of the factors on IFpH, viscosity, syneresis, color, organic and fatty acid profiles, and sensory properties was performed by one‐way and three‐factor randomized complete block design using SPSS 13.0 statistical software. The mean differences were analyzed using Tukey's multiple‐range test at 5% significance levels.

## 3 RESULTS AND DISCUSSION

### 3.1 Compositional analysis and physicochemical properties

After the first day of cold storage, the fat, total solids, ash, and protein contents of buffalo milk yogurt samples were 3.1 ± 0.0 g/100 g, 13.4 ± 0.2 g/100 g, 0.8 ± 0.0 g/100 g, and 4.2 ± 0.1 g/100 g, respectively. No significant (*p* > .05) effects of the SCT and IFpH) on the final compositions of the buffalo milk yogurt samples were observed since they were directly related with the composition of buffalo milk.

**Figure 2:**
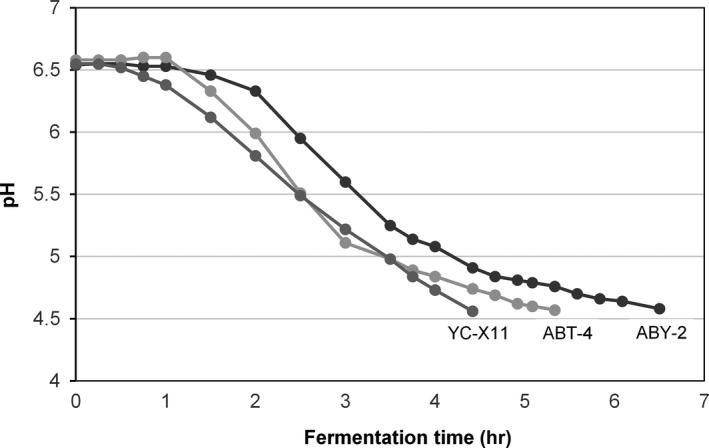
Changes in the pH during the fermentation of buffalo milk

The initial pH of the buffalo milk was standardized to reduce variability in the length of fermentation as stated by Nguyen et al. (2014). It was observed that the SCT primarily affected the acidification kinetic during fermentation (Figure 2). The fermentation time was prolonged by using probiotic ABY‐2 and ABT‐4 cultures. Regardless of the SCT, slight decreases in pH values were observed at the initial stages of all fermentations, which can be attributed to higher buffer capacity of buffalo milk (Medeiros, Souza, & Correia, 2015). A faster and steep curve inclination then occurred progressively. The effect of the SCT on the acidification kinetic was primarily important at the second stage of the fermentation. A third phase of the fermentation related to stabilization stated by Medeiros et al. (2015) was not observed with an exception of ABY‐2 culture in this study.

**Figure 3:**
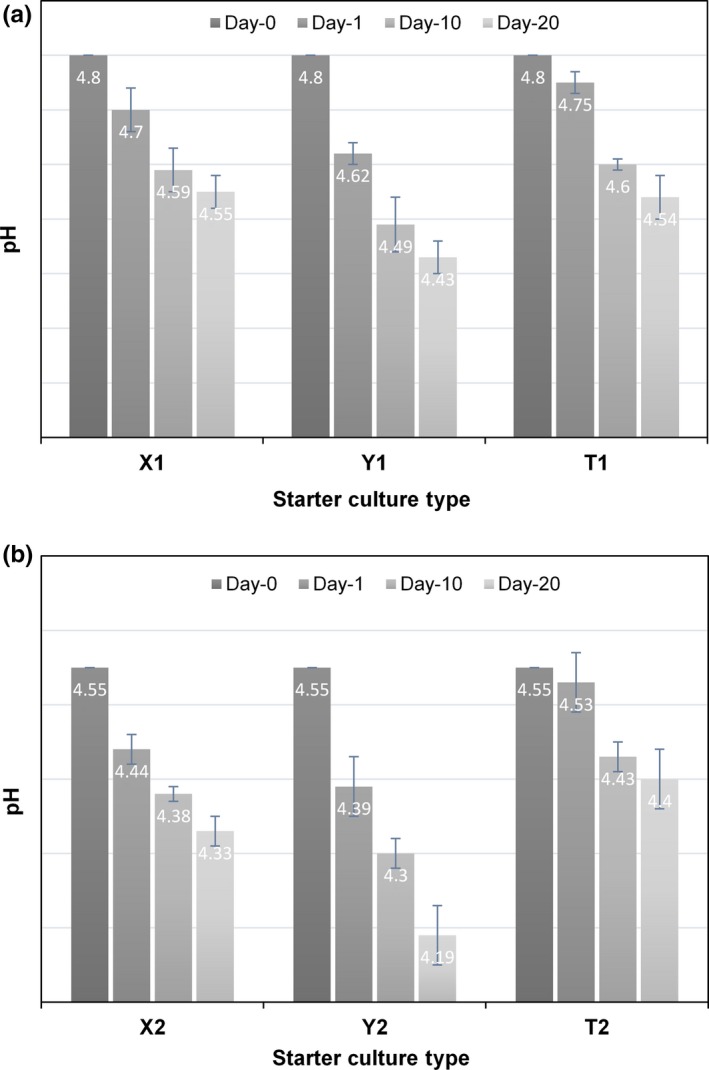
Changes in pH levels of buffalo milk yogurt samples during cold storage (incubation final pH of 4.80 [a]; incubation final pH of 4.55 [b])

As expected, the pH values of buffalo milk yogurt samples significantly declined throughout the cold storage (*p* < .05). For all buffalo milk yogurt samples, the IFpH did not affect the decline trend of pH during cold storage. The decrease in pH of the buffalo milk yogurt consisting of ABY‐2 culture was relatively higher than the buffalo milk yogurt samples consisting of YC‐X11 and ABT‐4 cultures (Figure 3). Regardless of the IFpH, buffalo milk yogurt fermented by ABY‐2 culture had the lowest pH after 20 days of cold storage, while the others exhibited similar trend.

Considering the whole period of shelf‐life, the titratable acidity increased with the postacidification during cold storage. As shown in Table 1, the SCT altered the postacidification kinetics of the buffalo milk yogurt samples during cold storage. When the IFpH was 4.80, titratable acidity was significantly affected by the SCT (*p* < .05). The increase in the titratable acidity fitted to zero‐order reaction for all buffalo milk yogurt samples during cold storage. However, the reaction rate constants differed due to the SCT for the IFpH of 4.80, while they were similar for the IFpH of 4.55. When the fermentation period lasted at the pH of 4.80, the reaction rate constant of postacidification of buffalo milk yogurt fermented by YC‐X11 was higher than the probiotic ones. The buffalo milk yogurt samples fermented by ABT‐4 had the lowest rate constant. On the other hand, when the fermentation period lasted at the pH of 4.55, the reaction rate constants of the buffalo milk yogurt samples fermented by probiotic strains increased, while the rate constant of the buffalo milk yogurt fermented by YC‐X11 remained nearly constant.

**Table 1:** The postacidification kinetics of the buffalo milk yogurt samples during cold storage

Yogurt sample	*n*	*k* (g·100 g^−1^·day^−1^)	*R* ^2^
X1	0	0.010	.99
Y1	0	0.007	.99
T1	0	0.004	.99
X2	0	0.009	.99
Y2	0	0.009	.99
T2	0	0.008	.99

*n*, reaction order; *k*, reaction rate constant.

In general, a symbiotic relationship between *S. thermophilus* and *L. delbrueckii* subsp. *bulgaricus* is valid during the processing of yogurt (Lourens‐Hattingh & Viljoen, 2001). During fermentation, *L. delbrueckii* subsp. *bulgaricus* produces essential amino acids which are necessary for the growth of *S. thermophilus*. *Streptococcus thermophilus* produces lactic acid simultaneously, which stimulates the growth of *L. delbrueckii* subsp*. bulgaricus*. As shown in Table 1, absence of *L. delbrueckii* subsp. *bulgaricus* in the ABT‐4 culture decreased the rate of postacidification. The stimulated growth of streptococci was responsible for the increased rate of postacidification, which can be attributed to *L. delbrueckii* subsp. *bulgaricus* presented in the ABY‐2 and YC‐X11, and fermentation period was lasted at the IFpH of 4.80. It was also stated that *S. thermophilus* acting as an oxygen scavenger creates an anaerobic environment and may enhance growth and survival of *Bifidobacterium* when used together in starter cultures (Lourens‐Hattingh & Viljoen, 2001). However, the similar trend was not observed in this study when the fermentation period was lasted at the IFpH of 4.55. It is well known that streptococci are inhibited at pH values of 4.2–4.4. At the low IFpH, lactobacilli are mainly responsible for the increase in acidity by producing excessive amounts of lactic acid. Differences occurred in kinetic behaviors of different starter cultures at different IFpH values may also depend on survival of probiotic bacteria in yogurt samples. Lourens‐Hattingh and Viljoen (2001) reported that final acidity and interaction between species present affected the survival of probiotic strains primarily during cold storage. It has also been reported that *L. acidophilus* survived better than the traditional yogurt culture organisms, *L. delbrueckii* subsp. *bulgaricus* and *S. thermophilus*, in yogurt under acidic conditions. On the other hand, most strains of *Bifidobacteria* are sensitive to pH values below 4.6. Lourens‐Hattingh and Viljoen (2001) stated that *L. delbrueckii* subsp. *bulgaricus* causes “overacidification” during storage and it can be prevented by using modified or ABT yogurt starter cultures. The kinetic results of the present study supported this statement when the fermentation period lasted at the IFpH of 4.80.

**Figure 4:**
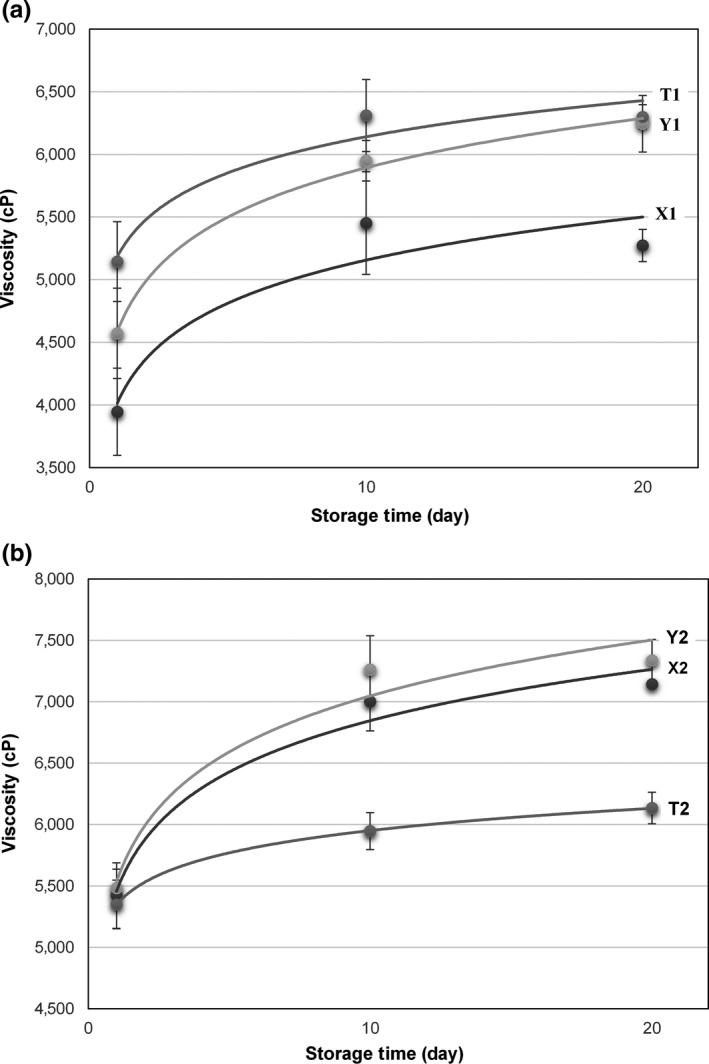
Changes in viscosity values of buffalo milk yogurt samples during cold storage (incubation final pH of 4.80 [a]; incubation final pH of 4.55 [b])

The IFpH did not affect the viscosity of T1 and T2. Also, the changes in viscosity values of T1 and T2 were similar during cold storage (Figure 4a,b). In T1 and T2, *S. thermophilus* was the sole fermenting organism. No symbiotic relationship exists during fermentation as *L. delbrueckii* subsp*. bulgaricus* is absent in the starter culture (Shihata & Shah, 2002). However, it was observed that the IFpH significantly affected the viscosity values of X1, X2, Y1, and Y2 (*p* < 0.05). Also, Y1 and Y2 had higher viscosity values than X1 and X2. The difference between YC‐X11 and ABY‐2 was maximum in the case of the IFpH of 4.80. The increment trend was also significantly different for X1, X2, Y1, and Y2 during cold storage (*p* < .05). Although pH changes in X1 and T1 were similar during cold storage, T1 had higher viscosity than X1 (*p* < .05). Shihata and Shah (2002) reported that proteolytic strains of *L. delbrueckii* subsp. *bulgaricus* are capable of hydrolyzing proteins and this may have led to reduction in viscosity of yogurt samples. In this study, it was similarly found that X1 and Y1 containing *L. delbrueckii* subsp. *bulgaricus* had lower viscosities than T1. Compared with the plain starter cultures and the probiotic ones, the viscosity of the buffalo milk yogurt sample increased as the fermentation time increased at the initial stage of cold storage (Figure 4a). The probiotic cultures prolonged the fermentation time increasing the viscosity of the buffalo milk yogurt samples when only at IFpH 4.80. Similar trend was not observed at the IFpH 4.55 (Figure 4b). Shihata and Shah (2002) correlated the increased firmness of the yogurt samples with incorporation of *L. delbrueckii* subsp. *bulgaricus* to ABT‐4 starter cultures. In our study, similar trend was observed at both IFpH 4.55 and 4.80 for the first day of the storage. The attachment of mucogenic strains to the protein matrix via the exopolysaccharide (the amount was not measured in this study) might be a reason for the improvement in viscosity. It was also indicated that faster exopolysaccharide production can be occurred when mixed cultures were used (Shihata & Shah, 2002).

The syneresis values of the buffalo milk yogurt samples containing YC‐X11 and ABY‐2 cultures ranged from 39.00% to 41.67%. The IFpH did not significantly affect the syneresis (*p* > .05). As shown in Table 2, the absence of *L. delbrueckii* subsp. *bulgaricus* in the culture increased the syneresis significantly (*p* < .05). In this case, it was also easily mentioned that there was no significant difference in T1 and T2 again (*p* > .05). Şanlı, Şenel, Sezgin, and Benli (2014) mentioned that the body of low‐fat yogurt could be improved using exopolysaccharide‐producing starter cultures. Although different conclusions are available in the literature (Badel, Bernardi, & Michaud, 2011) concerning the use of exopolysachharide in yogurt to improve rheology, the amount of starter culture, molecular characteristics of exopolysaccharides, and their ability to interact with protein network are known as important factors that affect the morphology of yogurt. Moreover, there are inconsistent data in the literature as the molecular characteristics of exopolysaccharides are also affected by the fermentation parameters (Mende, Rohm, & Jaros, 2016). Ibrahim (2015) also concluded that a longer fermentation time in camel milk leads to the formation of a weak structure. The main reasons for syneresis may be due to the structural rearrangements in casein micelles in the gel network and the rate of solubilization of colloidal calcium particles. In this study, a longer fermentation period was achieved by ABY‐2 culture. However, higher syneresis was observed in T1 and T2 including ABT‐4 culture (Table 2). Therefore, the primary reason for higher syneresis was considered to be the SCT instead of the fermentation period.

**Table 2:** Effect of different starter culture and incubation final pH on the syneresis value of buffalo milk yogurt samples during cold storage

Yogurt sample	Storage time (day)		
	1	10	20
X1	41.67 ± 3.06bcA	40.67 ± 0.58abAB	36.00 ± 2.00abB
X2	39.00 ± 2.00cA	36.67 ± 2.52bAB	34.00 ± 0.00bcB
Y1	40.33 ± 1.16cB	44.67 ± 0.58aA	34.67 ± 0.58bcC
Y2	39.00 ± 1.00cB	43.67 ± 2.08aA	31.67 ± 1.53cC
T1	45.67 ± 0.58abA	38.00 ± 1.00bB	36.33 ± 2.08abB
T2	46.67 ± 1.53aA	36.33 ± 2.31bB	39.33 ± 1.16aB

Means with same letters (A–C) in a row within the category data are not significant at *p* > .05.

Means with same letters (a–d) in a column within the category data are not significant at *p* > .05.

As shown in Table 2, a significant decrease in syneresis values of X1, X2, T1, and T2 was recorded after 20 days of cold storage (*p* < .05). Mahmood, Abbas, and Gilani (2008) reported similar trends in yogurt samples made with different commercial probiotic cultures. As it was indicated by Mende et al. (2016), the interaction between polysaccharide molecules and protein network is related to the acidity of the medium. It is generally assumed that polysaccharides that are directly attached to protein network are charged and acidity of a medium changes the charges of protein molecules. Repulsive or associative interactions or segregative phase separation can be occurred depending on pH and, thus, protein charge. Unlike the other buffalo milk yogurt samples, a significant increase was observed in the syneresis values of Y1 and Y2 after 10 days of cold storage (*p* < .05). The increase in syneresis might be related to the count of *L. acidophilus* as stated before (Olson & Aryana, 2008).

Whiteness (L*), red/greenness (a*), and yellow/blueness (b*) values of the buffalo milk yogurt samples are shown in Table 3. The mean L* value of buffalo milk yogurt samples was higher than that found in dahi (Raju & Pal, 2009), and was lower than that of found in probiotic yogurt (Olson & Aryana, 2008). L*, a*, and b* values of all buffalo milk yogurt samples were not significantly affected by the IFpH and the SCT (*p* > .05). It was also observed that 20 days cold storage did not significantly affect the L* values of X2, Y1, Y2, T1, and T2 (*p* > .05), and only the L* values of X1 (*p* < .05) were significant. Although the decrease in L* values of all the buffalo milk yogurt samples was observed in this study, these reductions were statistically insignificant (*p* > .05). The a* values of probiotic buffalo milk yogurt samples were closer to that found in a probiotic yogurt by Olson and Aryana (2008). As shown in Table 2, an irregular trend was observed in syneresis values of probiotic buffalo milk yogurt samples, which was attributed to the amount of syneresis during cold storage (Shirai et al., 1992), and this led to fluctuations in a* values of probiotic buffalo milk yogurt samples throughout the cold storage.

**Table 3:** Effect of different starter culture and incubation final pH on the color (L*, a*, and b*) of buffalo milk yogurt samples during cold storage

Physicochemical property	Yogurt sample	Storage time (day)		
		1	10	20
L* value	X1	85.45 ± 0.45abcA	85.34 ± 0.76bA	82.12 ± 1.00aB
	X2	84.50 ± 0.90cA	84.97 ± 0.21bA	82.11 ± 2.20aA
	Y1	84.77 ± 0.58bcA	84.98 ± 0.41bA	84.37 ± 0.90aA
	Y2	85.07 ± 0.25bcA	85.16 ± 0.10bA	85.28 ± 0.21aA
	T1	86.08 ± 0.08abA	86.73 ± 0.04aA	82.11 ± 4.29aA
	T2	86.53 ± 1.00aA	86.50 ± 0.09aA	86.23 ± 0.22aA
a* value	X1	−2.57 ± 0.50aA	−2.15 ± 0.86aA	−2.03 ± 0.19aA
	X2	−2.34 ± 0.62aA	−1.63 ± 0.75aA	−2.03 ± 0.44aA
	Y1	−2.71 ± 0.42aAB	−1.97 ± 0.51aA	−3.67 ± 0.22bB
	Y2	−2.28 ± 0.72aA	1.76 ± 0.25aA	−3.62 ± 0.06bB
	T1	−2.40 ± 0.13aA	−2.24 ± 0.49aA	−2.46 ± 0.96abA
	T2	−2.32 ± 0.31aAB	−1.76 ± 0.67aA	−3.00 ± 0.19abB
b* value	X1	14.44 ± 1.60aA	13.17 ± 2.49aA	13.27 ± 0.19bA
	X2	13.52 ± 2.09aA	11.45 ± 1.90aA	13.11 ± 0.60bA
	Y1	14.29 ± 1.74aAB	11.10 ± 1.51aB	17.70 ± 0.92aA
	Y2	12.37 ± 2.78aB	10.39 ± 0.78aB	17.94 ± 0.26aA
	T1	14.76 ± 0.10aB	13.78 ± 1.67aB	17.27 ± 0.34aA
	T2	14.47 ± 1.22aAB	12.11 ± 2.39aB	17.00 ± 0.92aA

Means with same letters (A–C) in a row within the category data are not significant at *p *>* *.05.

Means with same letters (a–d) in a column within the category data are not significant at *p *>* *.05.

The SCT and the IFpH did not significantly affect the b* values of all the buffalo milk yogurt samples (*p* > .05). Dimitreli, Petridis, Akakiadou, and Chrysalidou (2014) stated that the riboflavin content had higher importance on b* value of a yogurt sample than its composition. Olson and Aryana (2008) also reported that b* value of a yogurt sample was affected by the inoculation level of *L. acidophilus*, and higher inoculation level of *L. acidophilus* caused darker and more red and yellow yogurt. In this study, there were no significant differences in b* values of Y1, Y2, T1, and T2 even though ABY‐2 and ABT‐4 cultures contain *L. acidophilus* (*p* > .05) . However, the b* values of Y1, Y2, T1 and T2 were affected by the longer period of cold storage significantly (*p* < .05). The counts of probiotic microorganisms present in ABY‐2 and ABT‐4 cultures may be responsible for the increases in the b* values during cold storage.

### 3.2 Sensory evaluation

The changes in sensory properties of the buffalo milk yogurt samples during cold storage are shown in Table 4. It is clear that the IFpH did not significantly affect the flavor, texture, appearance, color, and general acceptability of all the buffalo milk yogurt samples (*p* > .05). The highest flavor and general acceptability scores were obtained for the yogurts made with ABY‐2 culture (*p* < .05). However, the effects of the SCT on the body, texture, appearance, and color of the buffalo milk yogurt samples were insignificant (*p* > .05). The flavor, body, texture, appearance, color, and general acceptability of Y1, Y2, T1, and T2 at 20 days of cold storage were not significant (*p* > .05). Cold storage slightly affected the flavor of X1 samples. Concurrently, X1 samples had the lowest flavor scores at 20 days of cold storage (*p* < .05).

**Table 4:** Effect of different starter culture and incubation final pH on the sensory properties of buffalo milk yogurt samples during cold storage

Sensory property	Yogurt sample	Storage time (day)		
		1	10	20
Flavor	X1	5.80 ± 1.81bB	8.00 ± 0.67aA	7.20 ± 2.10aAB
	X2	6.60 ± 1.90abA	7.10 ± 1.45aA	8.00 ± 1.83aA
	Y1	8.30 ± 1.49aA	7.10 ± 1.29aA	8.10 ± 1.20aA
	Y2	8.00 ± 1.41abA	7.80 ± 1.62aA	8.60 ± 1.51aA
	T1	6.80 ± 1.48abA	7.90 ± 1.29aA	7.60 ± 1.65aA
	T2	7.30 ± 2.11abA	7.30 ± 1.70aA	7.60 ± 1.08aA
Body and texture	X1	3.60 ± 1.27aA	4.40 ± 0.52aA	3.50 ± 0.85bA
	X2	4.30 ± 0.82aA	4.40 ± 0.52aA	5.10 ± 0.88abA
	Y1	4.40 ± 0.52aA	4.20 ± 1.03aA	4.70 ± 0.48aA
	Y2	4.40 ± 0.70aA	4.60 ± 0.70aA	4.60 ± 0.52aA
	T1	4.20 ± 0.79aA	4.10 ± 0.74aA	4.30 ± 0.68abA
	T2	4.60 ± 1.84aA	4.40 ± 0.70aA	4.40 ± 0.52aA
Appearance and color	X1	3.70 ± U6aA	4.20 ± 1.03aA	3.90 ± 1.10aA
	X2	4.00 ± 1.25aA	4.30 ± 1.06aA	4.40 ± 0.97aA
	Y1	4.50 ± 0.53aA	4.30 ± 0.68aA	4.60 ± 0.97aA
	Y2	4.00 ± 0.67aA	4.50 ± 0.71aA	4.60 ± 0.97aA
	T1	4.60 ± 0.70aA	4.90 ± 0.32aA	4.80 ± 0.63aA
	T2	4.80 ± 1.69aA	4.90 ± 0.32aA	4.80 ± 0.42aA
General acceptability	X1	6.10 ± 1.79bA	7.90 ± 0.99aA	7.10 ± 2.13aA
	X2	6.70 ± 1.49abA	7.40 ± 1.43aA	7.90 ± 1.79aA
	Y1	8.50 ± 1.35aA	7.30 ± 1.25aA	7.80 ± 1.03aA
	Y2	8.00 ± 1.76abA	8.00 ± 1.49aA	8.00 ± 1.49aA
	T1	7.30 ± 1.42abA	8.20 ± 1.14aA	7.60 ± 1.65aA
	T2	7.30 ± 1.83abA	7.20 ± 1.55aA	7.70 ± 1.06aA

Means with same letters (A–C) in a row within the category data are not significant at *p* > .05.

Means with same letters (a–d) in a column within the category data are not significant at *p* > .05.

Cunha Neto, Oliveira, Hotta, and Sobral (2005) used YC‐X11 culture for buffalo milk yogurt and reported that standardized buffalo milk yogurts containing 3 g/100 g fat obtained higher scores for taste in sensorial analysis at 15 and 30 days of storage. Similar result was observed in the flavor scores of the buffalo milk yogurts throughout the cold storage. The probiotic buffalo milk yogurt samples containing ABY‐2 and ABT‐4 cultures had higher general acceptability scores at 1 day of the cold storage, and longer storage time did not significantly affect the decision of the panelists anymore (*p* > .05).

The most noted defects by the panelists were insufficient acidity and plain taste for X1 and X2 at the 1st day of the cold storage. Thirteen percent of the panelists also indicated that X1 has rougher and grainier texture than the other buffalo milk yogurt samples.

### 3.3 Organic acid profile

In the following part of this study, organic acid and fatty acid contents of Y2 were analyzed and compared with X2, as the highest flavor and general acceptability scores were obtained for Y2 (*p* < .05) according to sensorial evaluation.

**Figure 5:**
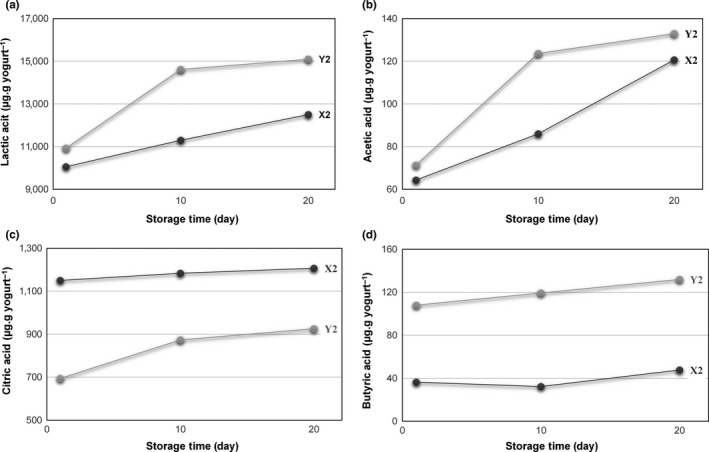
Changes in organic acids of buffalo milk yogurt samples during cold storage. (a) Lactic acid, (b) acetic acid, (c) citric acid, and (d) butyric acid

Lactic, acetic, citric, and butyric acids were identified as the main organic acids in X2 and Y2. The changes in the amount of these organic acids during cold storage are presented in Figure 5. Lactic acid and citric acid were the most abundant acids in Y2 and X2 samples during cold storage. Unlike X2, a little amount of formic acid, which might be due to the growth of *Bifidobacteria* as stated by Seckin and Ozkilinc (2011), was also detected in Y2 during cold storage (data not shown). Formic acid was not significantly affected by the storage time (*p* > .05). It was probably due to the limited growth of *Bifidobacteria* during cold storage. Adhikari, Grün, Mustapha, and Fernando (2002) also indicated that the metabolic inertness of the bifid bacterial cells can occur when they were added to the yogurt after fermentation.

It was found that the concentrations of lactic acid, acetic acid, and butyric acid in Y2 were higher than those of X2. The effect of the SCT on lactic acid and acetic acid production was not significant (*p* > .05). However, a significant difference was observed in butyric acid concentration (*p* < .05). Similarly, Donkor, Henriksson, Vasiljevic, and Shah (2006) reported that lactic acid concentration in yogurts containing probiotic culture was higher than the control containing standard yogurt culture. They also stated that the differences in lactic acid concentration were not statistically significant (*p* > .05). In contrast to the concentrations of other acids, it can be stated that X2 has higher amount of citric acid than Y2 (Figure 5c). Adhikari et al. (2002) noted that the citric acid content of the yogurts containing probiotic strains was lower than that of the control containing standard starter cultures. They related this result to the higher utilization of citric acid by the starter cultures during fermentation in the presence of bifid bacteria. As expected, the concentrations of organic acids significantly increased in both Y2 and X2 samples during cold storage with an exception of butyric acid (*p* < .05). Similarly, Adhikari et al. (2002) reported that there was no significant effect of the storage period on the concentration of butyric acid in set and stirred types of cow milk yogurt (*p* > .05). The SCT significantly affected the production of lactic acid, acetic acid, and citric acid during cold storage (Figure 5). The increases in the amounts of all organic acids presented in Figure 5 were found to be linear for X2 during 20 days of cold storage. However, for Y2, the amounts of lactic acid, acetic acid, and citric acid increased more rapidly in the first 10 days of cold storage. Then, the rate of increase slowed down gradually and further slight increases were observed throughout the storage period.

### 3.4 Fatty acid profile

The changes in concentrations of fatty acids in X2 and Y2 during cold storage are presented in Table 5. In both Y2 and X2 samples, the major fatty acids were myristic (C14:0), palmitic (C16:0), stearic (C18:0), and oleic (C18:1) acids. The ratio of total saturated fatty acids to total unsaturated fatty acids was calculated to be 58:42. Also, the effect of the SCT on the proportions of saturated and unsaturated fatty acids was not significantly important (*p* > .05). Yadav, Jain, and Sinha (2007) analyzed the control and probiotic dahi and reported the ratio of 65:35 and 55:45, respectively. The increase in unsaturated fatty acids of the buffalo milk yogurt samples might be mainly due to the increase in the amount of oleic acid (18:1) during fermentation. It was also easily noted that 20 days of cold storage did not significantly (*p* > .05) change the ratio of saturated fatty acids to unsaturated fatty acids. In general, the amounts of fatty acids in X2 and Y2 did not follow a regular trend during cold storage. Similar results were reported by Güler (2007), while Yadav et al. (2007) observed an increase in the amounts of fatty acids throughout the storage. Yadav et al. (2007) indicated that butyric acid was formed principally by lipolytic activity of lactic acid bacteria, and the lactobacilli having probiotic activity can produce conjugated linoleic acid (CLA) in milk during fermentation by lipolysis of natural milk fat. Alternatively, distinct changes in the butyric acid and linoleic acid contents of X2 and Y2 were not observed in this study, which can be attributed to the pH levels of the both Y2 and X2 samples. Kim and Liu (2002) also indicated that pH below 4.6 has more effect on CLA synthesis than SCT. Only, the decrease in linoleic acid content of X2 and Y2 samples was observed after 20 days of cold storage and it might be explained that the starter cultures used in this study utilized the linoleic acid as a substrate for CLA synthesis as stated by Yadav et al. (2007). Beshkova et al. (1998) stated that the hydrolytic activity of *S. thermophilus* toward milk fat is low, and the most important precursors of volatile fatty acids were amino acids. They also indicated that formation of volatile fatty acids (C2–C10) was more active in mixed cultures than that of in pure ones. However, SCT did not significantly affect the amounts of volatile fatty acids in this study (*p* > .05).

**Table 5:** Effect of starter culture types on the free fatty acids of buffalo milk yogurt samples during cold storage (mg/g fat)

Free fatty acid	Starter culture type	Storage time (day)		
		1	10	20
C_4:0_	X2	17.19 ± 0.54aA	16.93 ± 0.74aA	15.29 ± 0.06aB
	Y2	16.50 ± 0.09aB	16.13 ± 0.22aC	17.45 ± 0.20aA
C_6:0_	X2	7.13 ± 0.15aA	7.13 ± 0.06aA	6.78 ± 0.02aB
	Y2	7.09 ± 0.07aA	7.09 ± 0.11aA	7.08 ± 0.02aA
C_8:0_	X2	2.68 ± 0.05aA	2.73 ± 0.02aA	2.72 ± 0.01aA
	Y2	2.72 ± 0.03aA	2.74 ± 0.05aA	2.97 ± 0.40aB
C_10:0_	X2	4.95 ± 0.08aA	4.96 ± 0.03aA	4.97 ± 0.02aA
	Y2	4.98 ± 0.05aA	5.01 ± 0.06aA	5.02 ± 0.02aA
C_12:0_	X2	7.84 ± 0.09aA	7.80 ± 0.04aA	7.57 ± 0.05aB
	Y2	7.79 ± 0.03aA	7.84 ± 0.04aA	7.60 ± 0.04aB
C_13:0_	X2	1.43 ± 0.68aA	1.04 ± 0.01aA	1.02 ± 0.13aA
	Y2	1.03 ± 0.01aC	1.04 ± 0.01aB	1.16 ± 0.01aA
C_14:0_	X2	63.72 ± 0.46aB	63.06 ± 0.12aC	65.37 ± 0.33aA
	Y2	62.80 ± 0.28bA	62.82 ± 0.42aA	61.96 ± 0.15bB
C_14:1_	X2	5.21 ± 0.02aB	5.15 ± 0.03aC	7.86 ± 0.40aA
	Y2	5.16 ± 0.03aA	5.10 ± 0.03aB	5.13 ± 0.01bAB
C_15:0_	X2	22.40 ± 0.12aA	22.33 ± 0.05aA	21.50 ± 0.13aB
	Y2	22.26 ± 0.16aA	22.23 ± 0.07aA	21.64 ± 0.10aB
C_15:1_	X2	7.48 ± 0.02aA	7.51 ± 0.02aA	7.20 ± 0.04aB
	Y2	7.54 ± 0.04aA	7.50 ± 0.03aA	7.21 ± 0.05aB
C_16:0_	X2	303.30 ± 0.77aB	305.31 ± 0.23aA	287.13 ± 2.88aC
	Y2	305.29 ± 1.89aA	304.89 ± 1.05aA	296.24 ± 1.51aB
C_16:1_	X2	25.86 ± 0.06aA	25.85 ± 0.08aA	24.71 ± 0.19aB
	Y2	25.79 ± 0.17aA	25.65 ± 0.04aAB	25.57 ± 0.04aB
C_17:0_	X2	15.54 ± 0.06aB	15.66 ± 0.04aA	14.67 ± 0.16aC
	Y2	15.70 ± 0.08aA	15.61 ± 0.02aA	15.22 ± 0.07aB
C_17:1_	X2	8.47 ± 0.06aC	8.60 ± 0.09aB	8.96 ± 0.07aA
	Y2	8.57 ± 0.04aB	8.58 ± 0.08aB	8.86 ± 0.05aA
C_18:0_	X2	88.74 ± 0.46aB	89.89 ± 0.33aA	82.38 ± 1.17bC
	Y2	89.91 ± 0.74aA	89.77 ± 0.26aA	85.76 ± 0.69aB
C_18:1_	X2	316.33 ± 3.44aA	317.51 ± 1.89aA	313.20 ± 0.91aA
	Y2	317.25 ± 2.40aAB	316.38 ± 1.18aB	319.73 ± 0.67bA
C_18:2_	X2	12.43 ± 0.84aA	12.26 ± 0.06aA	10.79 ± 0.29aB
	Y2	12.32 ± 0.12aA	12.28 ± 0.05aA	11.61 ± 0.12aB
C_18:3_	X2	4.37 ± 0.04bC	4.46 ± 0.04aB	4.62 ± 0.04bA
	Y2	4.48 ± 0.06aB	4.44 ± 0.07aC	5.19 ± 0.12aA

Means with same letters (A–C) within the category data are not significant at *p *> .05.

Means with same letters (a–d) within the category data are not significant at *p *> .05.

## 4 CONCLUSIONS

The results of the presented study highlighted that the processing conditions such as the SCT and the IFpH altered the physicochemical and organoleptic properties of buffalo milk yogurt samples containing 3 g/100 g of milk fat. The IFpH mainly affected the activities of the strains in the mixed starter cultures. The probiotic cultures prolonged the fermentation time and decreased the reaction rate constant of postacidification during cold storage. Moreover, the extent of decrease in reaction rate constant varied depending on the IFpH. The absence of *L. delbrueckii* subsp. *bulgaricus* in the starter culture increased the viscosity for IFpH of 4.80. It also increased the syneresis for both IFpH 4.80 and 4.55. The IFpH did not affect the viscosity of T1 and T2, while it was vice versa for the other samples. Twenty days cold storage decreased the syneresis of the buffalo milk yogurt samples. L*, a*, and b* values of all the buffalo milk yogurt samples were not significantly affected by the IFpH and the SCT. The sensory results showed that a more acceptable buffalo milk yogurt was probiotic ABY‐2 culture added to yogurt samples. The organic acid and fatty acid profiles of the buffalo milk yogurt samples containing ABY‐2 culture were further analyzed and compared to the control yogurt containing YC‐X11 culture. The SCT significantly affected the production of lactic acid, acetic acid, and citric acid during cold storage. However, there was no significant effect of the SCT on the proportions of saturated and unsaturated fatty acids in the buffalo milk yogurt samples.

## CONFLICT OF INTERESTS

The authors declare that there is no conflict of interest regarding the publication of this paper.
